# A Novel Adaptively-Robust Strategy Based on the Mahalanobis Distance for GPS/INS Integrated Navigation Systems

**DOI:** 10.3390/s18030695

**Published:** 2018-02-26

**Authors:** Chen Jiang, Shu-Bi Zhang

**Affiliations:** 1 School of Environment Science and Spatial Informatics, China University of Mining and Technology, Xuzhou 221116, China; jiangchen@cumt.edu.cn; 2 Collaborative Innovation Center for Resource Utilization and Ecological Restoration of Old Industrial Base, China University of Mining and Technology, Xuzhou 221116, China

**Keywords:** adaptive filter, cubature Kalman filter, integrated navigation, Mahalanobis distance, robust estimation

## Abstract

As an optimal estimation method, the Kalman filter is the most frequently-used data fusion strategy in the field of dynamic navigation and positioning. Nevertheless, the abnormal model errors seriously degrade performance of the conventional Kalman filter. The adaptive Kalman filter was put forward to control the influences of model errors. However, the adaptive Kalman filter based on the predicted residuals (innovation vector) requires reliable observation information, and its performance is significantly affected by outliers in the measurements. In this paper, a novel adaptively-robust strategy based on the Mahalanobis distance is proposed to weaken the effects of abnormal model deviations and outliers in the measurements. In the proposed scheme, the judging index is defined based on the Mahalanobis distance, and the adaptively-robust filtering is performed when the observations are reliable, otherwise, the robust filtering is performed based on the robust estimation method. Various experiments with the actual data of GPS/INS integrated navigation systems are implemented to examine validity of the proposed scheme. Results show that both the influences of model deviations and outliers are weakened effectively by using the proposed adaptive robust filtering scheme. Moreover, the proposed scheme is easy to implement with a reasonable calculation burden.

## 1. Introduction

Since the properties of the Global Positioning System (GPS) and inertial navigation systems (INS) are complementary, the integration of these two systems has been extensively applied in the field of target tracking and navigation. As one of the most popular optimal estimators [1], the Kalman filter is the basic method of data fusion in many fields [2,3]. However, performance of the Kalman filter can be seriously degraded by outliers in the measurements or noise with a non-Gaussian distribution [4]. Consequently, an update of the priori information and compensation of the motion equations errors have become key problems [5]. Recently, various filtering algorithms and strategies have been proposed to control the influences of model deviations and perturbations in the measurements, such as the robust filter [6,7], H∞ filter [8,9], DIA (detection, identification and adaptation) methods [10], adaptive filter [11,12], and so forth. The robust filter is able to address both non-Gaussian distributed noises and outlying measurements, but the influence of model deviations cannot be controlled effectively. By minimizing the estimation error in the worst case, the H∞ filter can control the influences of uncertainties in the measurement noise [13], but it fails when outliers exist [7]. The DIA methods were put forward to control the influences of outliers of the integrated navigation systems. However, it is quite difficult to identify the model errors, especially when measurements are unreliable [14]. In practice, a variety of adaptive Kalman filters have been developed in different fields [11,12,15,16]. The function-model-based and the stochastic-model-based adaptive filter are two strategies of the adaptive filter, including the multiple-model-based adaptive estimation (MMAE) [11] and the innovation-based adaptive estimation (IAE), respectively [17]. The IAE strategy is more suitable for the GPS/INS integrated navigation systems [11], and it has been applied successfully in the GPS/INS integrated navigation systems.

Different from the MMAE and IAE strategies, Chinese researchers have developed an adaptively-robust filter by introducing the adaptive factors and robust estimation method [14]. Both adaptivity and robustness are provided in the proposed adaptively-robust filter, and it has been adopted successfully in many applications [18,19,20,21]. However, in the adaptively-robust filtering algorithm based on the differences between the estimated state parameters and predicted ones, the dimension of the state vector should be less than that of the measurements, thus, the algorithm can only be adopted in limited cases [4], and further investigation is required for the filter with both adaptivity and robustness. In general, the predicted residual series or the residual series is applied to update the covariance matrices of the adaptively-robust filter, and the predicted residual series should be adopted if thee dimension of the state vector is larger than that of the measurement vector, thus, the adaptively-robust algorithm can be applied in the GPS/INS integrated navigation systems. However, the predicted residual series is closely related to the measurements, and it would become unreliable once there exist outliers in the measurements, and the test statistic based on the predicted residual series would also be unreliable. Under this circumstance, performance of the adaptive filter may be degraded, or even inferior, than that of the conventional Kalman filter. Accordingly, an improved adaptively-robust strategy should be developed to control the influences of the outlying measurements.

In this paper, an improved adaptively-robust filter is proposed based on the adaptive Kalman filter, robust estimation method and Mahalanobis distance. In the proposed strategy, the Mahalanobis distance is adopted as error detection statistics, and it determines whether the adaptive strategy is performed or not. Two cases are considered in this paper, and contrastive experiments and analysis are performed with the data collected by the GPS/INS integrated navigation systems under real conditions. The obtained results show that an improved adaptively-robust strategy manifests better performance; meanwhile, it is easily implemented.

The remainder of this paper is organized as follows: In Section 2, basic principles of the adaptive filter and the adaptively-robust filter are introduced. In Section 3, an improved adaptively-robust strategy is proposed and the process of the proposed comprehensive filtering algorithm is concluded. In Section 4, basic theory of the GPS/INS integrated navigation systems are displayed, and the dynamic and measurement models for the loosely-coupled GPS/INS integrated navigation systems are introduced. Section 5 demonstrates the advantages of the proposed filtering strategy through actual experiments. The conclusions are given in Section 6.

## 2. Adaptive Filter and Adaptively-Robust Filter

### 2.1. Basic Principles of Adaptive Filter

The adaptive filter has been widely applied and investigated in the field of dynamic navigation and positioning. Different from the Sage-Husa filter [11], a new series of adaptive filters were developed by constructing single or multiple adaptive factors [20]. 

For the dynamic and measurement equations:

(1) $$\left\{ \begin{array}{l} x_{k}\;=\;\Phi_{k,k-1}x_{k-1}\;+\;w_{k}\\ z_{k}\;=\;H_{k}x_{k}\;+\;v_{k} \end{array}\right.,$$

where $x_{k}$ denotes the state vector, $\Phi_{k,k-1}$ denotes the transition matrix, $z_{k}$ denotes the measurement vector, $H_{k}$ denotes the measurement matrix, $w_{k}$ and $v_{k}$ denote the system state noise and the measurement noise, respectively. The process of the time update is given by:

(2) $$x_{k/k-1}\;=\;\Phi_{k,k-1}x_{k-1},$$

(3) $$P_{k/k-1}\;=\;\Phi_{k,k-1}P_{k-1}\Phi^{T}{}_{k,k-1}\;+\;Q_{k},$$

where $x_{k/k-1}$ denotes the predicted state vector, $P_{k/k-1}$ denotes the predicted covariance matrices of $x_{k/k-1}$, and $Q_{k}$ denotes the covariance matrix of $w_{k}$. With the least squares (LS) estimation, the extremum condition is defined by:

(4) $$V_{k}^{T}R_{k}^{-1}V_{k}+V_{x_{k/k-1}}^{T}P_{k/k-1}^{-1}V_{x_{k/k-1}}=\min,$$

where $V_{k}$ and $V_{x_{k/k-1}}$ denote the residuals of $z_{k}$ and $x_{k/k-1}$, respectively. Thus, the solution of the conventional Kalman filter is obtained by:

(5) $$K_{k}=P_{k/k-1}H_{k}^{T}{\left(H_{k}P_{k/k-1}H_{k}^{T}+R_{k}\right)}^{-1},$$

(6) $$x_{k/k}=x_{k/k-1}+K_{k}(z_{k}-H_{k}x_{k/k-1}),$$

where $R_{k}$ denotes the covariance matrix of measurement noise. With the adaptive factor $\alpha_{k}$ and the LS estimation, the extremum condition can be redefined by:

(7) $$V_{k}^{T}R_{k}^{-1}V_{k}+\alpha_{k}V_{x_{k/k-1}}^{T}P_{k/k-1}^{-1}V_{x_{k/k-1}}=\min.$$

Thus, solution of the adaptive Kalman filter is obtained by:

(8) $$x_{k/k}={\left(H_{k}^{T}R_{k}^{-1}H_{k}+\alpha_{k}P_{k/k-1}^{-1}\right)}^{-1}(H_{k}^{T}R_{k}^{-1}z_{k}+\alpha_{k}P_{k/k-1}^{-1}x_{k/k-1}),$$

and the iterative solution is presented by:

(9) $${\bar{K}}_{k}=\frac{1}{\alpha_{k}}P_{k/k-1}H_{k}^{T}(\frac{1}{\alpha_{k}}H_{k}P_{k/k-1}H_{k}^{T}+R_{k}),$$

(10) $$x_{k/k}=x_{k/k-1}+{\bar{K}}_{k}(z_{k}-H_{k}x_{k/k-1}),$$

(11) $$P_{k}=P_{k/k-1}-{\bar{K}}_{k}H_{k}P_{k/k-1},$$

where ${\bar{K}}_{k}$ denotes the equivalent gain matrix.

### 2.2. Adaptive Factor

The differences between conventional filters and adaptive filters originate from the adaptive factor. Consequently, to find a suitable adaptive factor becomes the key problem in the adaptive filter design. In recent years, four types of test statistic and adaptive factors have been proposed [20]. The predicted residual is calculated based on the predicted state of the current epoch, and it is not modified by current measurements. Consequently, the predicted residual is more suitable to express perturbations of the dynamic system.

Since the test statistic constructed by the predicted residual is applied in this paper, only this type of statistic is displayed. The test statistic based on the predicted residual is defined by:

(12) $${\bar{V}}_{k}=H_{k}x_{k/k-1}-z_{k},$$

(13) $$\Delta {\bar{V}}_{k}={\left(\frac{{\bar{V}}_{k}^{T}{\bar{V}}_{k}}{\sqrt{tr(P_{{\bar{V}}_{k}})}}\right)}^{\frac{1}{2}},$$

(14) $$P_{{\bar{V}}_{k}}=H_{k}P_{k/k-1}H_{k}^{T}+R_{k},$$

where ${\bar{V}}_{k}$ denotes the predicted residual, $\Delta {\bar{V}}_{k}$ denotes the error detection statistic, and $P_{{\bar{V}}_{k}}$ denotes the covariance matrix of the predicted residual. It should be noticed that the error detection statistic $\Delta {\bar{V}}_{k}$ is closely related to the predicted residual ${\bar{V}}_{k}$, and ${\bar{V}}_{k}$ is determined by the observation information at the current epoch. Thus, the reliable measurements are required to achieve a qualified statistic.

The test statistic and the adaptive factor constructed based on the predicted residual need no redundant measurements, and they are applicable when the number of measurements is less than that of the unknown parameters, accordingly, they are suitable for the GPS/INS integrated navigation systems. The adaptive factors are constructed by the test statistic. For instance, the two-segment adaptive factor is defined by [20]:

(15) $$\alpha_{k}=\left\{ \begin{array}{ll} 1 & \left|\Delta {\bar{V}}_{k}\right|\leq c\\ \frac{c}{\left|\Delta {\bar{V}}_{k}\right|} & \left|\Delta {\bar{V}}_{k}\right|>c \end{array}\right.,$$

where c (1.0 ≤ c ≤ 1.5) denotes the threshold. In general, c is determined by experience. It is demonstrated that the adaptive factor is inversely proportional to the test statistic, but it does not decrease to zero. Similar to the IGGш equivalent weight function [22,23], the three-segment adaptive factor based on the predicted residual is defined by:

(16) $$\alpha_{k}=\left\{ \begin{array}{ll} 1 & \left|\Delta {\bar{V}}_{k}\right|\leq c_{0}\\ \frac{c_{0}}{\left|\Delta {\bar{V}}_{k}\right|}{\left(\frac{c_{1}-\left|\Delta {\bar{V}}_{k}\right|}{c_{1}-c_{0}}\right)}^{2} & c_{0}<\left|\Delta {\bar{V}}_{k}\right|\leq c_{1}\\ 0 & \left|\Delta {\bar{V}}_{k}\right|>c_{1} \end{array}\right..$$

where $c_{0}$ and $c_{1}$ are the criteria fixed by experience, and $1.0\;\leq \;c_{0}\;\leq \;1.5$, $3.0\;\leq \;c_{1}\;\leq \;8.5$. In the three-segment adaptive factor, the factor would become zero if $\left|\Delta {\bar{V}}_{k}\right|$ is greater than $c_{1}$. It should be noticed that in Equations (9) and (10) $\alpha_{k}\;\neq \;0$. However, there exist other adaptive factors that are not displayed here.

### 2.3. The Adaptively-Robust Filter

Although the influences of model deviations can be controlled with the adaptive filter, the adaptive factor cannot resist the influences of the outlying measurements. Consequently, the robust estimation method was adopted to develop the adaptively-robust filtering algorithm [14].

For the adaptively-robust filtering algorithm, the extremum condition is defined by [20]:

(17) $$\sum_{i=1}^{n_{k}}p_{i}\rho (v_{i})+\alpha_{k}{\left({\hat{x}}_{k}-{\hat{x}}_{k/k-1}\right)}^{T}P_{k/k-1}({\hat{x}}_{k}-{\hat{x}}_{k/k-1})=\min,$$

where $p_{i}$ denotes the *i*th diagonal element of the weight matrix, and ρ denotes the continuous non-minus convex function [22]. The solution of the adaptively-robust filter is obtained according to the Equation (17):

(18) $$x_{k/k}=x_{k/k-1}+{\bar{K}}_{k}(z_{k}-H_{k}x_{k/k-1}),$$

(19) $${\bar{K}}_{k}=\frac{1}{\alpha_{k}}P_{k/k-1}H_{k}^{T}(\frac{1}{\alpha_{k}}H_{k}P_{k/k-1}H_{k}^{T}+{\bar{R}}_{k}),$$

where ${\bar{R}}_{k}$ denotes the equivalent covariance matrix of the measurement noise.

The adaptively-robust filtering algorithm can resist the effects of state perturbations and outlying measurements simultaneously. Moreover, it shows a noticeable flexibility because it can automatically select the most suitable algorithm from LS estimation, Kalman filter, robust filter, adaptive filter, and adaptively-robust filter [20].

## 3. A Novel Adaptively-Robust Filtering Strategy

As mentioned above, estimates of the unknown parameters are closely related to the measurements, thus, the performance of the adaptive filter based on the predicted residual is determined by the quality of the measurements. With the outlying measurements, the predicted residual becomes unreliable and, consequently, the performance of the conventional Kalman filter may even be degraded by the adaptive factor. Namely, the adaptive factor may backfire with the unreliable residuals. Thus, the adaptive factor should be applied in a more suitable way, and an improved adaptively-robust strategy is proposed below.

For the system defined by Equation (1), the measurements should be Gaussian distributed with the mean ${\bar{z}}_{k}$ and covariance $P_{{\bar{z}}_{k}}$ to achieve an optimal solution, and the probability density function $\rho (z_{k})$ is defined by [4]:

(20) $$\rho (z_{k})=N(z_{k};{\bar{z}}_{k},P_{{\bar{z}}_{k}})=\frac{\exp\left(-\frac{1}{2}{\left(z_{k}-{\bar{z}}_{k}\right)}^{T}{\left(P_{{\bar{z}}_{k}}\right)}^{-1}(z_{k}-{\bar{z}}_{k})\right)}{\sqrt{{\left(2\pi \right)}^{m}\left|P_{{\bar{z}}_{k}}\right|}},$$

where m denotes the dimension of a measurement vector. However, if the measurement’s noise does not obey the Gaussian distribution, or some outlying measurements exist, the probability density function would no longer hold. On the other hand, if the probability density function does not hold, the Gaussian distribution of measurement noise is contaminated, or some outliers exist. Accordingly, this property can be applied to perform the hypothesis test to detect the abnormal perturbations in the measurements.

In fact, the test statistic can be constructed according to the probability density function and the covariance $P_{{\bar{z}}_{k}}$ is the a priori assumption. Moreover, in the actual applications, the dimension of a measurement vector is known. Thus, the square of the Mahalanobis distance from $z_{k}$ to ${\bar{z}}_{k}$ is adopted as a test statistic, namely:

(21) $$M_{k}^{2}={\left(z_{k}-{\bar{z}}_{k}\right)}^{T}{\left(P_{{\bar{z}}_{k}}\right)}^{-1}(z_{k}-{\bar{z}}_{k}),$$

where $M_{k}$ denotes the Mahalanobis distance. The Mahalanobis distance is a kind of covariance distance of the data and it is scale-invariant. In theory, the square of the Mahalanobis distance should be Chi-square distributed with the freedom degree r, and the Mahalanobis distance has been applied to make the Kalman filter robust [4]. Under a confidence level α and freedom degree r, the α-quantile is $\chi_{\alpha }$. If $M_{k}^{2}$ is larger than $\chi_{\alpha }$, the null hypotheses should be rejected and it is concluded that some outlying measurements exist, then the key problem is to control the influences of the outlying measurements. Otherwise, the null hypotheses should be accepted and no abnormal measurements exist.

In this paper, the robust estimation method is applied to address the problem of outlying measurements. For the robust estimation method presented in this paper, the equivalent covariance matrix constructed based on the double-factor is adopted, and the scaling factor $\lambda_{ij}$ based on the predicted residual is defined by [24]:

(22) $$\lambda_{ij}=\sqrt{\lambda_{ii}\cdot \lambda_{jj}},$$

(23) $$\lambda_{ii}=\left\{ \begin{array}{ll} 1 & \left|{\bar{V}}_{{\bar{X}}_{k_{i}}}\right|\leq c\\ \frac{\left|{\bar{V}}_{{\bar{X}}_{k_{i}}}\right|}{c} & \left|{\bar{V}}_{{\bar{X}}_{k_{i}}}\right|>c \end{array}\right.,$$

where $\lambda_{ii}$ is one of the double-factors and $\lambda_{jj}$ can be obtained in the same way as $\lambda_{ii}$, $\left|{\bar{V}}_{{\bar{X}}_{k_{i}}}\right|$ is the element of the standardized predicted residual, and 1.0≤c≤1.5. Then, the equivalent covariance matrix (${\bar{R}}_{k}$) of the measurement noise is given by:

(24) $${\bar{R}}_{k}\;=\;\lambda_{ij}R_{k}.$$

**Figure 1:**
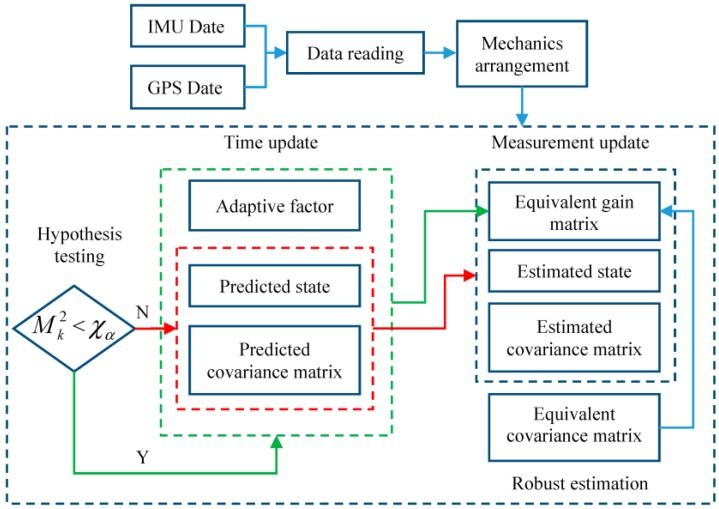
Flowchart of the novel adaptively-robust filtering strategy.

It is concluded that both the adaptive factor $\alpha_{k}$ and the scaling factor $\lambda_{ij}$ are constructed through the predicted residual ${\bar{V}}_{k}$. Influences of outlying measurements on the filtering results can be controlled by the robust estimation, however, the influences on the adaptive factor cannot be controlled, and this may cause noticeable effects on the filtering results. Consequently, the improved adaptively-robust filtering strategy is constructed. In the improved adaptively-robust filtering strategy, the hypothesis test is performed, first, at all epochs, and the testing results determine the following strategy. Only the robust estimation method is performed if test statistic $M_{k}^{2}$ is smaller than $\chi_{\alpha }$, otherwise, the robust estimation method is applied together with the adaptive factor. Namely, the adaptive filter is performed only at the epochs wherein the tested measurements do not contain outliers. Figure 1 displays the process of the proposed filtering strategy.

## 4. GPS/INS Integrated Navigation Systems

Three types of coupling have been developed for the GPS/INS integrated navigation systems, and the loosely-coupled system is designed in this paper. Considering the deviations of the position $\Delta R^{e}$, the velocity $\Delta V^{e}$ under the Earth-centered and Earth-fixed coordinate (e frame), the attitude error $\varphi^{e}$, and drift of the gyroscope $\nabla^{b}$ and accelerometer $\varepsilon^{b}$ under the body frame (b frame), the state vector $\hat{X}$ in this paper is defined by:

(25) $$\begin{array}{ll} \hat{X} & =[\Delta R^{e}\;\Delta V^{e}\;\varphi^{e}\;\nabla^{b}\;\varepsilon^{b}]\\ & =[\delta x,\;\delta y,\;\delta z,\;\delta v_{x},\;\delta v_{y},\;\delta v_{z},\;\delta \phi_{e},\;\delta \phi_{n},\;\delta \phi_{u},\;\delta g_{x},\;\delta g_{y},\;\delta g_{z},\;\delta a_{x},\;\delta a_{y},\;\delta a_{z}] \end{array}.$$

For the INS system, the nonlinear differential error model is defined by [25]:

(26) $$\left\{ \begin{array}{l} \Delta {\dot{R}}^{e}=\Delta V^{e}\\ \Delta {\dot{V}}^{e}=(I_{3\times 3}-C_{e^{\prime }}^{e})f^{e^{\prime }}+C_{b}^{e^{\prime }}\nabla^{b}-2\Omega_{ie}^{e}\Delta V^{e}\\ {\dot{\varphi }}^{e}=(I_{3\times 3}-C_{e}^{e^{\prime }})\omega_{ie}^{e}-C_{b}^{e^{\prime }}\varepsilon^{b}\\ {\dot{\nabla }}^{b}=0\\ {\dot{\varepsilon }}^{b}=0 \end{array}\right.,$$

where the symbol “˙” denotes the derivation, $I_{3\times 3}$ denotes the three-dimensional unit matrix, $C_{e^{\prime }}^{e}$ denotes the rotation matrix between e and $e^{\prime }$ frame, $C_{b}^{e^{\prime }}$ denotes the rotation matrix between b frame and $e^{\prime }$ frame, and $\Omega_{ie}^{e}$ denotes the skew symmetric matrix of the Earth rotation rate $\omega_{ie}^{e}$.

In general, dynamic models of the GPS/INS integrated navigation systems are nonlinear, thus, the Kalman filter cannot be applied directly. As such, the cubature Kalman filter is adopted to address the nonlinear problem in this paper.

f(⋅) and h(⋅) are assumed to be the known nonlinear functions, and the discrete nonlinear system is given by:

(27) $$\left\{ \begin{array}{l} x_{k}=f(x_{k-1})+w_{k}\\ z_{k}=h(x_{k})+v_{k} \end{array}\right..$$

The iterative equations of the cubature Kalman filter are listed below [26]:

(a) Time update
  (28) $$\left\{ \begin{array}{l} s_{k-1/k-1}=SVD(P_{k-1/k-1})\\ X_{k-1,k-1}=s_{k-1/k-1}\xi +x_{k-1/k-1}\\ X_{k/k-1}^{*}=f(X_{k-1,k-1}) \end{array}\right.,$$
  (29) $$x_{k/k-1}=\frac{1}{m}\sum_{i=1}^{m}X_{i,k/k-1}^{*},$$
  (30) $$P_{k/k-1}=\frac{1}{m}\sum_{i=1}^{m}X_{i,k/k-1}^{*}X_{i,k/k-1}^{*T}-x_{k/k-1}x_{k/k-1}^{T}+Q_{k}.$$
(b) Measurement update
  (31) $$K_{k}=P_{xz,k/k-1}P_{zz,k/k-1}^{-1},$$
  (32) $$x_{k/k}=x_{k/k-1}+K_{k}(z_{k}-z_{k/k-1}^{}),$$
  (33) $$P_{k/k}=P_{k/k-1}^{}-K_{k}P_{zz,k/k-1}K_{k}^{T}.$$

Assume that s is the square root of P, $X_{k-1,k-1}$ are the cubature points for the states vector, m is the number of the cubature points, and m=2n, n is the dimension of the state vector, $X_{k/k-1}^{*}$ are the propagated cubature points, $Z_{k/k-1}$ is the propagated cubature points for the measurement vector, and the corrected equations are given by:

(34) $$s_{k/k-1}=SVD(P_{k/k-1}),$$

(35) $$X_{k/k-1}=s_{k/k-1}\xi +x_{k/k-1},$$

(36) $$P_{k/k}=P_{k/k-1}^{}-K_{k}P_{zz,k/k-1}K_{k}^{T}.$$

(37) $$Z_{k/k-1}=h(X_{i,k/k-1}),$$

(38) $$z_{k/k-1}=\frac{1}{m}\sum_{i=1}^{m}Z_{i,k/k-1},$$

(39) $$P_{xz,k/k-1}=\frac{1}{m}\sum_{i=1}^{m}X_{i,k/k-1}Z_{i,k/k-1}^{T}-x_{k/k-1}z_{k/k-1}^{T},$$

(40) $$P_{zz,k/k-1}=\frac{1}{m}\sum_{i=1}^{m}Z_{i,k/k-1}Z_{i,k/k-1}^{T}-z_{k/k-1}z_{k/k-1}^{T}+R_{k}.$$

The position and velocity differences between the GPS and INS are the measurement inputs in the loosely-coupled systems. $r_{\mathrm{GPS}}$, $v_{\mathrm{GPS}}$, $r_{\mathrm{INS}}$, and $v_{\mathrm{INS}}$ are assumed to be the position and velocity of the GPS and INS, respectively, and the measurement vector $z_{k}$ is given by:

(41) $$z_{k}=\left[\begin{array}{l} r_{\mathrm{GPS}}-r_{\mathrm{INS}}\\ v_{\mathrm{GPS}}-v_{\mathrm{INS}} \end{array}\right].$$

Obviously, the measurement equation is linear in the loosely-coupled system, thus, the measurement equation should be rewritten by:

(42) $$\left\{ \begin{array}{l} x_{k/k}=x_{k/k-1}+K_{k}(z_{k}-H_{k}x_{k/k-1})\\ K_{k}=P_{k/k-1}H_{k}^{T}{\left(H_{k}P_{k/k-1}H_{k}^{T}+R_{k}\right)}^{-1}\\ P_{k/k}=P_{k/k-1}-K_{k}H_{k}P_{k/k-1} \end{array}\right..$$

In the context of multi-sensor fusion, the Kalman filter is the most commonly-used fusion method. As shown in Equation (42), estimation results are connected with the quality of the measurements, and imprecise measurements may result in large deviations of the estimates. Accordingly, the imprecise measurements should be addressed carefully. In the GNSS/INS integrated navigation systems, the adaptive factor and the robust estimation method constructed by the predicted residual can be adopted to improve the filtering performance [20].

## 5. Testing and Analysis

Various contrastive experiments were designed and performed. Data in these experiments were collected in real environments by a vehicle equipped with the GPS/INS integrated navigation systems. The integrated systems were composed of two GPS receivers (a base station and a rover station) and a low-cost SPAN-CPT IMU (inertial measurement unit). Table 1 displays the main technological parameters of IMU. The initial error of the position was 1.0 m, and initial error of the velocity was 0.1 m/s. The GPS output information on position and velocity were obtained by the double difference pseudorange measurements with a variances of (0.5 m)^2^ and (0.05 m/s)^2^. The sampling frequencies of GPS and INS were 1 Hz and 100 Hz, respectively. The results of a double difference carrier were used as references.

Experiments were composed of two cases, and four schemes were designed for each case. All the experiments were implemented based on the cubature Kalman filter. Results of the references were regarded as a true value, and the differences between the results of each scheme and references were regarded as errors. The robust estimation method presented in this paper are all applied based on the double-factor. The four schemes were designed as follows:

Scheme 1: the cubature Kalman Filter (CKF);
Scheme 2: the adaptive filter performed at all epochs (AKF-ALL) (two-segment function was adopted in the adaptive filter and c=1.0);
Scheme 3: the adaptive filter performed at the epochs without outliers (AKF-PARTIAL) (two-segment function was adopted in the adaptive filter and c=1.0);
Scheme 4: the improved adaptively-robust filter (IARF) (two-segment function was adopted in the adaptive filter and c=1.0).

(1) Case 1

**Figure 2:**
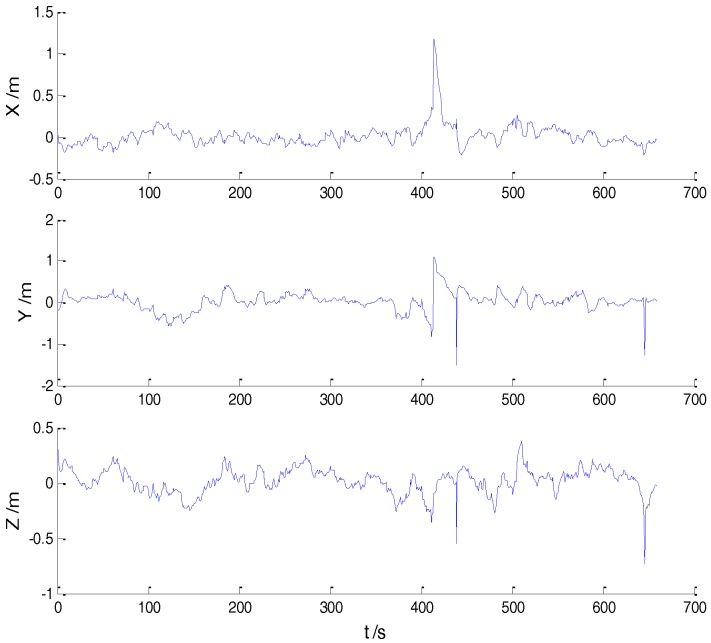
Position errors of the CKF scheme.

**Figure 3:**
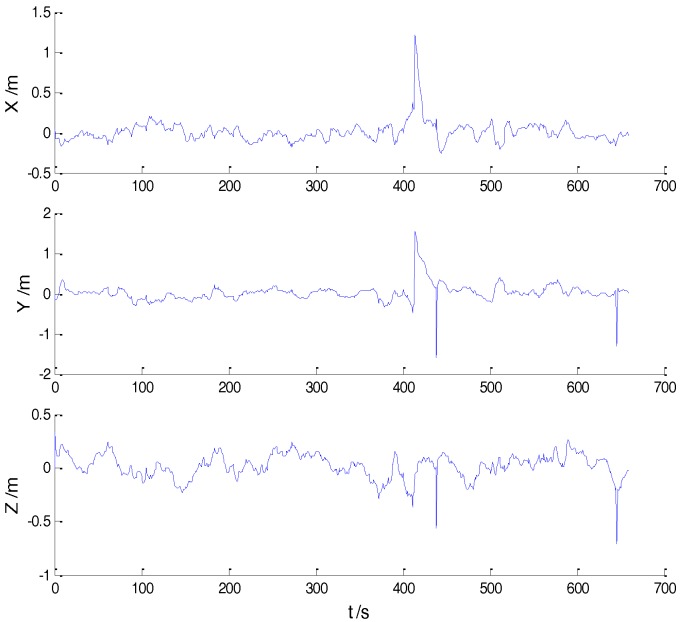
Position errors of the AKF-ALL scheme.

**Figure 4:**
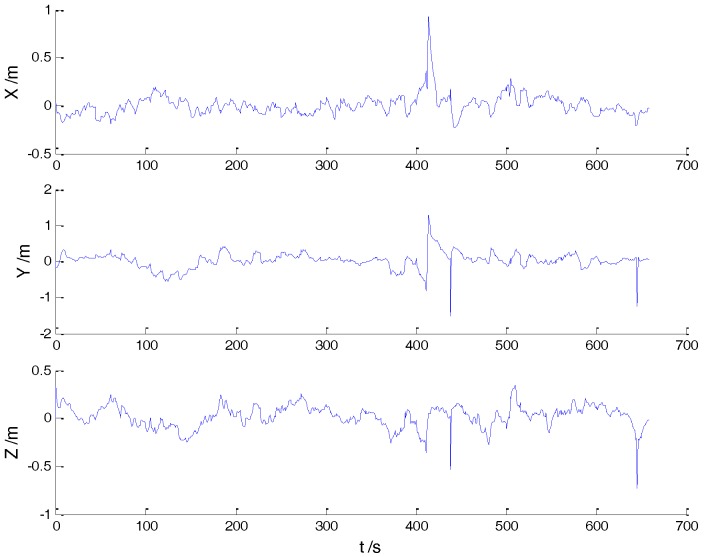
Position errors of the AKF-PARTIAL scheme.

**Figure 5:**
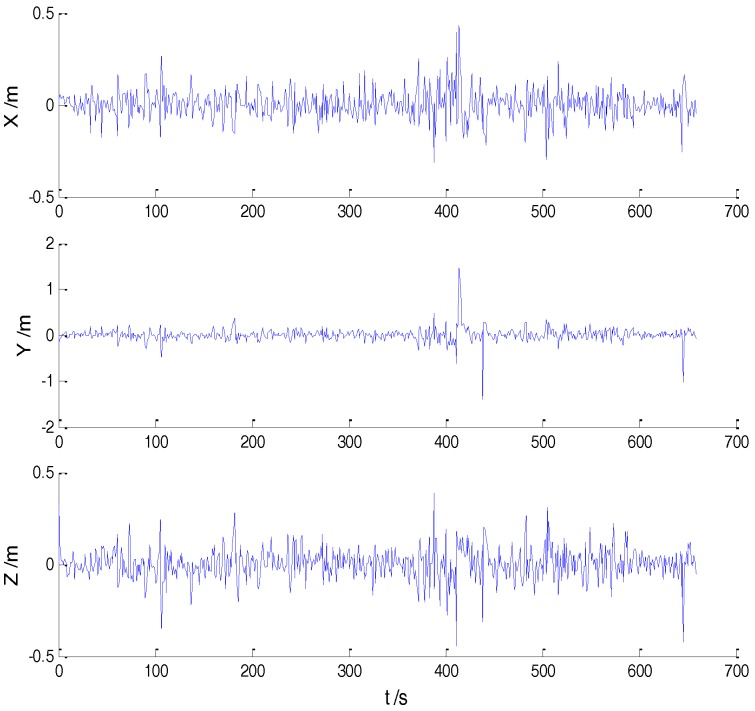
Position errors of the IARF scheme.

In Case 1, four schemes were implemented based on the initial measurements. Errors of the position in *x*, *y*, and *z* directions of each scheme are displayed in Figure 2, Figure 3, Figure 4 and Figure 5:

In the initial measurements, the filter performance was mainly affected by abnormal deviations of the dynamic model and effects of the outlying measurements are negligible. When the vehicle was passing through the speed hump, the abnormal perturbations might occur and filter precision could be degraded. It is obvious that there exist abnormal perturbations in Figure 2, Figure 3 and Figure 4, which indicates that robustness of both cubature Kalman filter and adaptive filter can be enhanced further. Since the test statistic constructed by the Mahalanobis distance is smaller than the threshold at most epochs, results of the AKF-ALL scheme and the AKF-PARTIAL scheme are similar. Compared with the AKF-PARTIAL scheme, the AKF-ALL scheme performed the adaptive filter at all epochs and effects of the dynamic model errors were well controlled. Figure 5 demonstrates that position errors in three directions of the IARF scheme were smaller than those of the other schemes, thus, a better performance was obtained with the improved adaptively-robust strategy.

To compare the performance of each scheme in a clearer way, the root mean square error (RMSE) of the position errors in three directions for each scheme were calculated and they are listed in Table 2.

As displayed in Table 2, both AKF-ALL and AKF-PARTIAL schemes manifested better performances than the CKF scheme, which indicates that influences of the dynamic model errors were weakened. Comparing results of AKF-ALL and AKF-PARTIAL schemes, it can be concluded that the latter suffered a slightly more influences of the dynamic model errors since the adaptive filter was not implemented at some epochs. By integrating the advantages of the adaptive filter, the robust estimation method, and the hypothesis test with the Mahalanobis distance, the adaptive filter was well performed and the effects of the dynamic model errors and outlying measurements were well controlled. Additionally, the RMSE of the IARF scheme were much smaller than those of the other schemes, which denotes a higher filtering precision.

(2) Case 2

**Figure 6:**
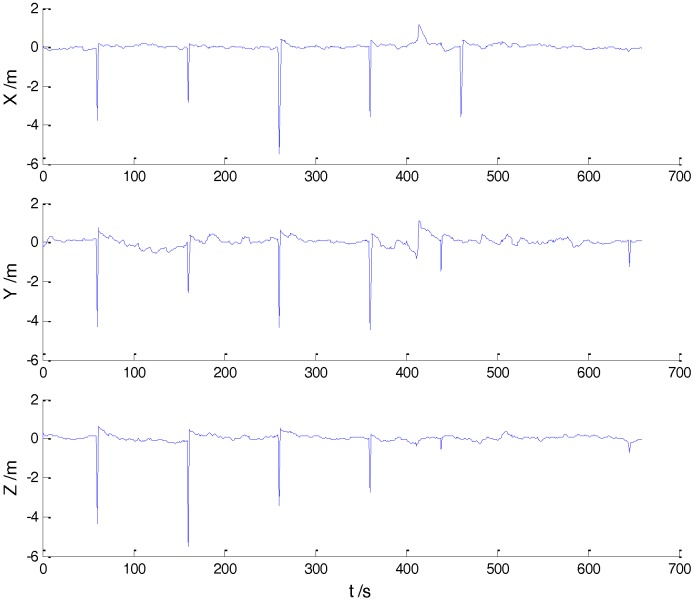
Position errors of the CKF scheme.

**Figure 7:**
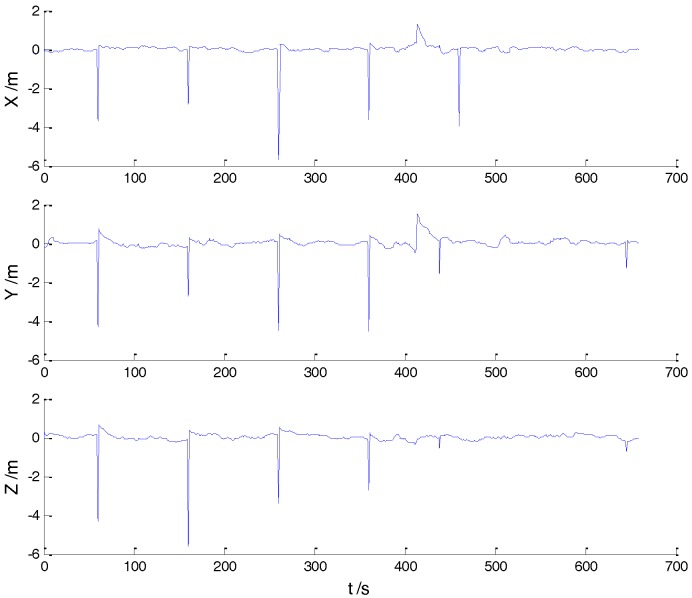
Position errors of the AKF-ALL scheme.

**Figure 8:**
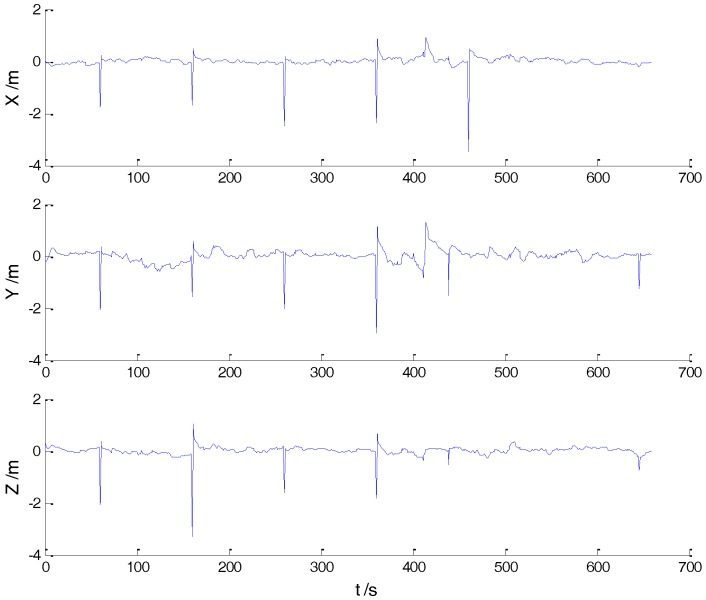
Position errors of the AKF-PARTIAL scheme.

**Figure 9:**
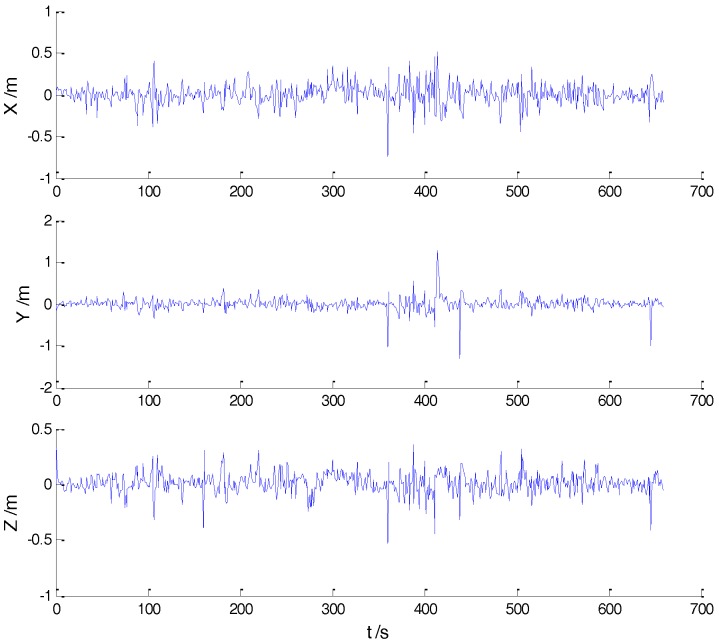
Position errors of the IARF scheme.

In order to test the robustness of the improved adaptively-robust strategy, the scattered outliers were added artificially to the initial measurements, thus, the data with outliers were constructed. Then, the aforementioned four schemes were performed with the data contained by the outlying measurements. The position errors of all schemes are displayed in Figure 6, Figure 7, Figure 8 and Figure 9:

Since outliers were added into the measurements, the filter performance was affected mainly by them. Obviously, Figure 6, Figure 7 and Figure 8 show that both CKF and AKF schemes manifested a low ability to resist the influences of the outliers, and the filtering results were affected significantly. Error amplitudes of the CKF scheme and AKF-ALL scheme are similar. Since the predicted residuals were contaminated in some epochs, deviations brought through the adaptive factor were introduced into the filter. Consequently, the AKF-ALL scheme performed only slightly better, or even inferior, than the CKF scheme. Compared with the first two schemes, influences of the dynamic model errors were weakened and influences of the outliers were not inflated with the AKF-PARTIAL scheme, thus, the error amplitude in each direction was decreased. However, performance of the AKF-PARTIAL scheme was still seriously affected by outliers because the outliers were not addressed effectively. Since the effects of the dynamic model errors and outlying measurements were controlled simultaneously, the IARF scheme exerted a better performance and the error amplitudes were much smaller.

The RMSE of the position errors in three directions for each scheme are listed in Table 3.

As can be seen in Table 3, in line with the error amplitudes, the RMSE of the CKF scheme and AKF-ALL scheme are similar, and it is concluded that performance of the CKF scheme was similar, or even better, than that of the AKF-ALL scheme under the influences of the outlying measurements. Both AKF-PARTIAL and IARF schemes performed better than the first two schemes. Moreover, the precision of the IARF scheme was much higher than those of the other schemes.

## 6. Conclusions

In this paper, an improved adaptively-robust filter is proposed based on the adaptive filter, robust estimation method, and Mahalanobis distance, and a new filtering strategy is developed. The proposed strategy is tested with the data collected by the GPS/INS integrated navigation systems in real circumstances. The detailed conclusions are summarized as follows:

(1) Comparing the performance of different algorithms in this paper, it can be summarized that the adaptive filter is able to control the influences of dynamic model errors, but it cannot resist the influences of outlying measurements, thus, the performance of the Kalman filter may be degraded by the adaptive factor when outlying measurements exist.
(2) Different from the conventional adaptively-robust filter, the adaptive factor of the new filtering strategy is performed according to the Mahalanobis distance. The proposed filtering strategy is verified in the loosely-coupled GPS/INS integrated navigation systems, and the robustness is demonstrated with both initial data and perturbative data.
(3) Comparing two cases, precision improvements are more obvious with the outlying measurements, thus, it is concluded that the proposed filtering strategy is more applicable when system is contaminated with outlying measurements.

## Figures and Tables

**Table 1:** Main technological parameters of the IMU.

Sensors	Random Bias	Random Constant Noise
Gyroscope	20 (°)/h	0.0667 (°)${/h}^{1/2}$
Accelerometer	5 mg	50 ${\mu g/h}^{1/2}$

**Table 2:** RMSE of schemes (m).

Axis	CKF	AKF-ALL	AKF-PARTIAL	IARF
X	0.130	0.117	0.121	0.096
Y	0.230	0.212	0.226	0.145
Z	0.118	0.114	0.116	0.084

**Table 3:** RMSE of schemes (m).

Axis	CKF	AKF-ALL	AKF-PARTIAL	IARF
X	0.373	0.380	0.248	0.123
Y	0.399	0.390	0.289	0.149
Z	0.355	0.354	0.221	0.104
